# Expression of the Novel Cardiac Biomarkers sST2, GDF-15, suPAR, and H-FABP in HFpEF Patients Compared to ICM, DCM, and Controls

**DOI:** 10.3390/jcm9041130

**Published:** 2020-04-15

**Authors:** Peter Jirak, Rudin Pistulli, Michael Lichtenauer, Bernhard Wernly, Vera Paar, Lukas J. Motloch, Richard Rezar, Christian Jung, Uta C. Hoppe, P. Christian Schulze, Daniel Kretzschmar, Rüdiger C. Braun-Dullaeus, Tarek Bekfani

**Affiliations:** 1 Clinic of Internal Medicine II, Department of Cardiology, Paracelsus Medical University of Salzburg, 5020 Salzburg, Austria; m.lichtenauer@salk.at (M.L.); b.wernly@salk.at (B.W.); v.paar@salk.at (V.P.); l.motloch@salk.at (L.J.M.); r.rezar@salk.at (R.R.); u.hoppe@salk.at (U.C.H.); 2 Division of Vascular Medicine, Department of Cardiology and Angiology, University Hospital Muenster, Albert-Schweitzer-Campus 1, Munster, North Rhine-Westphalia, 48149 Münster, Germany; Rudin.Pistulli@ukmuenster.de; 3 Division of Cardiology, Pulmonology, and Vascular Medicine, Medical Faculty, University Duesseldorf, 40225 Duesseldorf, Germany; Christian.Jung@med.uni-duesseldorf.de; 4 Department of Internal Medicine I, Division of Cardiology, Angiology, Pneumology and Intensive Medical Care, University Hospital Jena, Friedrich Schiller University Jena, 07740 Jena, Germany; Christian.Schulze@med.uni-jena.de (P.C.S.); daniel.kretzschmar@med.uni-jena.de (D.K.); 5 Department of Internal Medicine I, Division of Cardiology, Angiology and Intensive Medical Care, University Hospital Magdeburg, Otto von Gericke University, Magdeburg, 39120 Magdeburg, Germany; r.braun-dullaeus@med.ovgu.de (R.C.B.-D.);

**Keywords:** HFpEF, heart failure, HFrEF, biomarker, sST2, suPAR, H-FABP, sST2

## Abstract

Background: Heart failure with preserved ejection fraction (HFpEF) remains an ongoing therapeutic and diagnostic challenge to date. In this study we aimed for an analysis of the diagnostic potential of four novel cardiovascular biomarkers, GDF-15, H-FABP, sST2, and suPAR in HFpEF patients compared to controls as well as ICM, and DCM. Methods: In total, we included 252 stable outpatients and controls (77 DCM, 62 ICM, 18 HFpEF, and 95 controls) in the present study. All patients were in a non-decompensated state and on a stable treatment regimen. Serum samples were obtained and analyzed for GDF-15 (inflammation, remodeling), H-FABP (ischemia and subclinical ischemia), sST2 (inflammation, remodeling) and suPAR (inflammation, remodeling) by means of ELISA. Results: A significant elevation of GDF-15 was found for all heart failure entities compared to controls (*p* < 0.005). Similarly, H-FABP evidenced a significant elevation in all heart failure entities compared to the control group (*p* < 0.0001). Levels of sST2 were significantly elevated in ICM and DCM patients compared to the control group and HFpEF patients (*p* < 0.0001). Regarding suPAR, a significant elevation in ICM and DCM patients compared to the control group (*p* < 0.0001) and HFpEF patients (*p* < 0.01) was observed. An AUC analysis identified H-FABP (0.792, 95% CI 0.713–0.870) and GDF-15 (0.787, 95% CI 0.696–0.878) as paramount diagnostic biomarkers for HFpEF patients. Conclusion: Based on their differences in secretion patterns, novel cardiovascular biomarkers might represent a promising diagnostic tool for HFpEF in the future.

## 1. Introduction

With an overall prevalence of 2%, heart failure (HF) represents one of the leading causes of morbidity and mortality in the western world and thus also an important economic factor [1]. About 50% of all heart failure patients suffer from heart failure with preserved ejection fraction (HFpEF). HFpEF is characterized by a deterioration of cardiac relaxation resulting in an impaired diastolic filling of the left ventricle, mainly triggered by arterial hypertension along with obesity and metabolic disorders [2,3]. In contrast to heart failure with reduced ejection fraction (HFrEF), the left ventricular ejection fraction in HFpEF remains preserved [2,3]. 

The cellular processes involved in the development of HFpEF are heterogeneous. One of the most generally accepted hypotheses is that cellular hypertrophy combined with a reduction in cellular relaxation and an increase in tissue fibrosis could contribute strongly to the development of ventricular stiffening [4,5]. Furthermore, obesity, which is a very frequent co-morbidity of HF, leads to adipose tissue dysfunction along with elevated leptin levels and can trigger an upregulation of aldosterone, leading to sodium retention [6]. In consequence, higher levels of aldosterone trigger a volume expansion leading to increased filling pressures, thereby promoting cardiac remodeling, myocardial hypertrophy and fibrosis [6]. 

While numerous advancements have been made in the pharmacologic treatment of heart failure with reduced ejection fraction over the last decades (e.g., ARNIs), no evidence-based therapy for HFpEF patients exists to date [3,7]. Despite huge efforts, studies failed to show a significant prognostic benefit of pharmaceutical therapies in HFpEF, with the “PARAGON-Trial” as most prominent example [8]. Accordingly, the prognosis in HFpEF remains poor [9]. 

In addition to the lack of an evidence-based therapy, the actual diagnosis of HFpEF remains challenging and the precise diagnostic criteria are still matter of ongoing debates [9]. According to the current ESC guidelines, HFpEF is defined as a combination of: (I) Typical signs and symptoms of heart failure, (II) elevated levels of natriuretic peptides, (III) LVEF > 50%, (IV) evidence of diastolic dysfunction and/or structural heart disease (left ventricular hypertrophy or left atrial enlargement) [3]. Given the vague diagnostic criteria, the need for novel and additional diagnostic markers for HFpEF is evident.

In the last years, novel cardiac biomarkers have emerged as promising diagnostic tools for the assessment of different cardiovascular disease entities [10,11]. As a result to the complex pathophysiological background of most cardiovascular diseases, a multi-marker approach was reported as most effective for diagnosis, therapy monitoring and risk prediction due to the incorporation of different pathophysiologic processes covered by each respective marker [10,12].

Among the tested markers in previous studies, H-FABP (myocardial ischemia), sST-2 (myocardial strain and inflammation), GDF-15 (inflammation, remodeling), and suPAR (inflammation, remodeling) proved to be promising tools in achieving an improvement in the diagnosis and prognosis of cardiovascular diseases [13,14,15,16]. Accordingly, some of the listed markers are already included in the current guidelines and used in clinical routine [17]. 

Given the evident need for novel diagnostic tools in HFpEF we aimed for a head-to-head analysis of these four novel cardiovascular biomarkers in patients with heart failure with preserved ejection fraction compared to controls. Additionally, as the aforementioned markers are well studied in HFrEF patients, we aimed for a head-to-head analysis of HFpEF and HFrEF patients to put our findings into reference.

## 2. Experimental Section

The present study was conducted in accordance with the Universal Declaration of Helsinki and was approved by the local ethics committee at the University Hospital Jena, Germany. In total, we included 252 patients in this retrospective single-center study. Seventy-seven patients diagnosed with DCM, 62 patients with ICM, and 18 patients diagnosed with HFpEF were enrolled. Additionally, a control group of 95 patients was included. In these patients, coronary artery disease was excluded by coronary angiography. During visits in the outpatient ward, serum samples of all patients were obtained and analyzed for GDF-15, H-FABP, sST2, and suPAR.

The diagnosis of ICM, DCM and HFpEF was made according to the current guidelines of the European Society of Cardiology [3]. Clinical examination, assessment of medical history, laboratory analysis as well as transthoracic echocardiography was performed in all patients in the outpatient ward. Additionally, ICM and DCM patients underwent coronary angiography for diagnosis/exclusion of coronary artery disease. Controls also underwent coronary angiography because of suspected coronary artery disease and a relevant risk profile (hypertension, smoking etc.) and evidenced a rule out. All patients were in a stable, non-decompensated state at the timepoint of inclusion and clinical examination and were on a stable treatment regimen. Decompensated HF patients were not enrolled in this study. All examinations were performed by an experienced heart failure specialist. Laboratory analysis was conducted in all patients after informed consent. Serum samples were analyzed by means of ELISA and were stored at −80°C until measurements were conducted. Exclusion criteria were defined as: (I) Age under 18 years, (II) acute or chronic infections, (III) malignancies, (IV) advanced stages of renal failure (as indicated by a glomerular filtration rate less than 30 mL/min), (V) decompensated heart failure, (VI) hyperthyroidism, (VII) medication with immunosuppressive agents, and (VIII) recent acute coronary syndrome. For HFpEF patients a glomerular filtration rate under 60 ml/min was an exclusion criterion to rule out a potential cardiorenal confounder in this cohort.

### 2.1. Laboratory Analysis

Routine analysis of blood samples was performed at the Department of Clinical Chemistry (University Hospital Jena). The analyses comprised high-density lipoprotein (HDL; mmol/L), low density, lipoprotein (LDL; mmol/L), triglycerides (mmol/L), and C-reactive protein (CRP, mg/L) and hematological parameters. The glomerular filtration rate was calculated according to the CKD-EPI equation. Serum levels of sST2, GDF-15, suPAR, and H-FABP were measured using commercially available ELISA kits (DuoSet ELISA, DY523B, DY957, DY807, DY1678, and DFTA00, R&D Systems, Minneapolis, Minnesota, USA) in accordance with the instructions provided by R&D. ELISA analyses were performed at room temperature. In brief, 96-well plates were coated with the provided capture antibody according to the certificate of analysis and manufacturer’s instructions. The multiwell plates were incubated overnight on a horizontal shaker. The next day, plates were washed using 0.5% Tween 20 (Carl Roth, Karlsruhe, Germany) in 1× phosphate buffered saline (PBS) and were then blocked with 1% bovine serum albumin (BSA; Carl Roth, Karlsruhe, Germany) in 1× PBS for one hour. After a further washing step, serum and the appropriate standard concentrations for sample quantification were added onto the wells and incubated for two hours. Again, the plate was washed and the provided biotin-labelled detection antibody was added to each well, followed by an incubation of another two hours. Thereafter, ELISA plates were washed again, before a provided streptavidin-horseradish-peroxidase (HRP) solution was added and incubated for 20 min. After a final washing step, the addition of the substrate tetramethylbenzidine (TMB; Sigma Aldrich, St. Louis, Missouri, USA) resulted in a blue color reaction which was stopped by adding 2 N sulfuric acid (H_2_SO_4_; Sigma Aldrich, St. Louis, Missouri, USA), changing the color to yellow. Optical density (OD) was measured at 450 nm on an ELISA microplate reader (iMark Microplate Absorbance Reader, Bio-Rad Laboratories, Wien Austria).

### 2.2. Statistical Analysis

Statistical analysis was performed using GraphPad-Prism software (GraphPad-Software, La Jolla, CA, USA), SPSS (22.0, SPSS Inc., Chicago, IL, USA) and MedCalc (19.1.3 MedCalc Software bv, Ostend, Belgium). The Kolmogorov-Smirnov test was used to assess normal distribution of parameters in the study population. Demographic parameters were compared by using ANOVA. Normally distributed parameters are given as mean + standard deviation. As biomarker concentrations were not normally distributed, they are given as median and inter-quartile range. Median values were compared using the Mann–Whitney-U test. Correlation analysis was performed using Spearman’s rank-coefficient. Correction for multiple comparison was conducted using the Bonferroni–Holm method. ROC analysis was performed and AUCs were compared according to DeLong [18]. A *p* < 0.05 was considered as statistically significant.

## 3. Results

### 3.1. Baseline Characteristics

In total, the present study included 252 patients with a mean age of 62.6 years. While the distribution of male and female patients was quite balanced in HFpEF patients and controls, the HFrEF collective showed a significant higher number of male patients (*p* < 0.001). HFpEF patients were considerably older, compared to ICM, DCM, and controls (*p* < 0.001). Ejection fraction was significantly higher in patients with HFpEF compared to ICM and DCM patients (*p* < 0.001). BNP levels were significantly elevated in ICM (*p* < 0.001) and DCM (*p* < 0.001) compared to controls and HFpEF, while renal function was significantly impaired in the HFrEF collective (*p* < 0.001).

Regarding comorbidities, the rates of diabetes were evenly distributed in all three heart failure entities. Hypertension was present in similar rates in controls, HFpEF and ICM patients, with DCM patients showing significantly lower rates (*p* < 0.001). The rates of atrial fibrillation were significantly increased in HFpEF patients compared to all other entities (*p* < 0.001). With regards to medical therapy, HFrEF patients evidenced significantly higher rates beta-blockers, ACE-inhibitors and diuretics compared to HFpEF and controls (*p* < 0.001). Similarly, the rates of aldosterone antagonists were also higher in the HFrEF collective compared to HFpEF and controls (*p* < 0.001). Baseline characteristics are depicted in Table 1 and Table 2

### 3.2. Biomarkers

**Figure 1:**
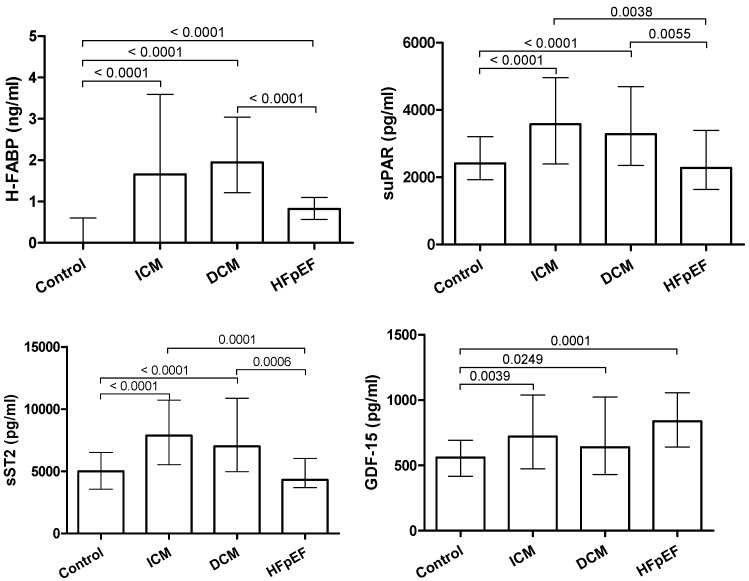
Comparison of biomarker levels between control group, HFpEF, ICM, and DCM patients (median + IQR).

GDF-15, evidenced a significant elevation for all heart failure entities compared to controls (*p* < 0.005) with no significant differences between the respective groups. For H-FABP, a significant elevation in all heart failure entities was observed compared to the control group (*p* < 0.0001). However, H-FABP levels were significantly higher in ICM and DCM patients compared to HFpEF (*p* < 0.0001). Levels of sST2 were significantly higher in ICM and DCM patients than in the control group (*p* < 0.0001). No significant differences between HFpEF patients and the control group were observed for sST2. Similar to sST2, levels of suPAR were significantly elevated in ICM and DCM patients compared to the control group (*p* < 0.0001) and HFpEF patients (*p* < 0.01). No significant differences between HFpEF patients and controls were observed. Biomarker levels are depicted in Table 3, comparisons of biomarker levels are depicted in Figure 1. In addition, a correction for multiple comparison was conducted by using the Bonferroni–Holm method. After correction for multiple testing, we found no changes in the statistical significance of our findings except for GDF-15 levels in controls vs. DCM. Correlation analysis of baseline characteristics and biomarkers of are given in the supplement Table S1. Results after multiple testing are given in the supplement Table S2. All biomarkers evidenced a significant correlation with BNP, Creatinine and CRP as well as an inverse correlation with ejection fraction.

### 3.3. AUC-Analysis

**Figure 2:**
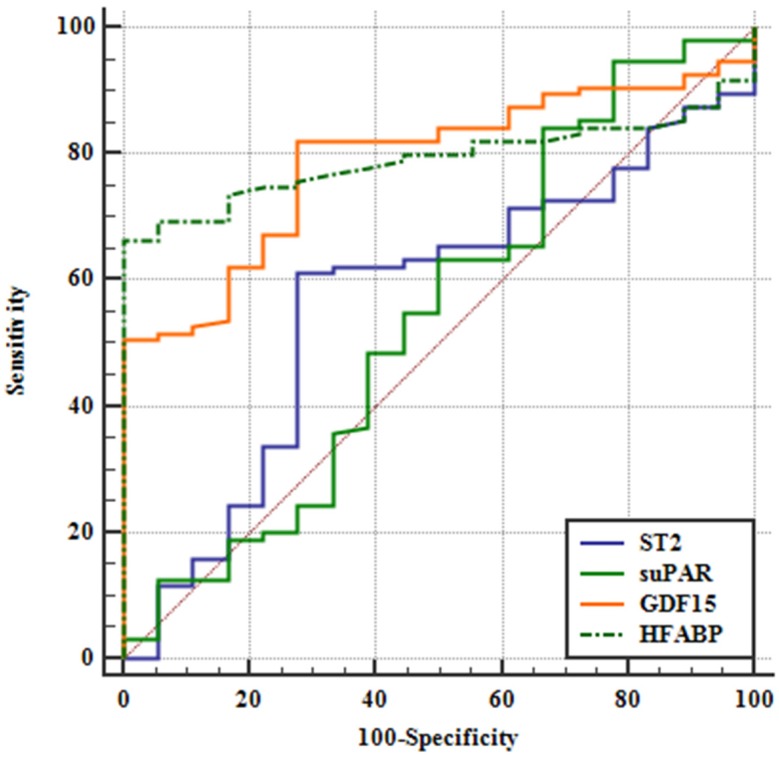
Receiver operating curve.

To evaluate the diagnostic potential of tested biomarkers in HFpEF, a ROC analysis was performed (Figure 2), and AUC was calculated for sST2, suPAR, GDF-15 and H-FABP plasma levels as diagnostic indicators for HFpEF patients. Our analysis identified H-FABP (0.792, 95% CI 0.713–0.870) and GDF-15 (0.787, 95% CI 0.696–0.878) as paramount diagnostic biom markers. In comparison, sST2 (0.567, 95%CI 0.294–0.572) and suPAR (0.543, 95% CI 0.298–0.616) evidenced a considerably lower AUC. The detailed results are depicted in Table 4. Additionally, we conducted a pairwise comparison of ROC curves according to DeLong et al. [18]. Here GDF-15 and H-FABP showed significantly higher AUCs compared to sST2 and suPAR respectively, while no significant difference between GDF-15 and H-FABP was observed. The detailed results are depicted in Table 5.

## 4. Discussion

Despite the growing awareness, HFpEF remains a diagnostic and clinical challenge to date. This is partially related to its complex pathophysiology [9]. Given the increasing prevalence of HFpEF and the high rates of misdiagnosis, the need for new diagnostic tools is evident [5]. Accordingly, we aimed for a head-to-head analysis of four novel cardiovascular biomarkers and their diagnostic benefit in patients with HFpEF compared to controls to address this evident gap. 

Regarding baseline characteristics we observed significant differences between the respective patient collectives. HFpEF patients were the oldest subgroup in our study, a finding that is typical for this disease entity and also matches former studies. A slow progression of myocardial fibrosis and remodeling with gradual diastolic impairment might explain the delayed onset of symptoms and consequently the higher age. Additionally, ICM and DCM patients evidenced worse renal function as well as decreased ejection fraction and significantly elevated BNP levels compared to HFpEF and controls. Moreover, HFpEF patients evidenced lower rates of a standard heart failure therapy, a finding which must be mainly attributed to the lack of an evidence-based therapy for HFpEF patients.

With regards to levels of GDF-15, a significant elevation was present in all three types of heart failure compared to controls. HFpEF patients provided the highest levels in the study collective, however without significant differences between HFpEF in comparison to HFrEF patients. While the detailed mechanisms involved in the GDF-15 pathway are not yet fully understood, it seems to be involved in the regulation of apoptosis, cell repair, and cell growth [15,19]. Accordingly, latest studies have also demonstrated a correlation between GDF-15 and atrial and myocardial fibrosis along with a prognostic impact in cardiovascular disease [20,21]. Additionally, GDF-15 is also involved in the regulatory processes of inflammatory pathways [22]. GDF-15 levels were shown to be significantly elevated in HFrEF in former studies [10]. However, the finding of an increase in GDF-15 in HFpEF patients represents a new aspect. The elevation might be attributed to the progressive myocardial fibrosis and remodeling involved in this disease entity, which could act as a trigger for the secretion of GDF-15. As GDF-15 has shown a significant prognostic impact in HFrEF patients, a similar prognostic value can be assumed for HFpEF patients. As potential surrogate for fibrosis burden, GDF-15 might also act as a monitoring parameter for HFpEF patients in the future.

H-FABP represents a highly sensitive marker for myocardial ischemia [23]. We observed a significant increase in all three heart failure entities. For HFrEF patients, an increase in H-FABP was reported in earlier studies and subclinical myocardial ischemia was proposed as the most probable cause for this finding [10]. Interestingly, based on our results it seems that subclinical ischemia is also present in HFpEF patients. A possible explanation might be a relative shortage in myocardial oxygen supply, based on various processes such as increased wall thickness of the left ventricle in this group of patients. Above all, due to the impaired ventricular filling, a relative shortage in blood supply is present [4]. Moreover, ventricular hypertrophy primarily triggered by arterial hypertension might add to this shortage [4]. Nevertheless, former studies have also shown a considerable prevalence of storage diseases such as amyloidosis and Morbus Fabry resulting in HFpEF [24]. Additionally, also an impairment in coronary microcirculation by means of coronary microvascular endothelial inflammation increasing resting tension through a reduction in nitric oxide bioavailability, cyclic guanosine monophosphate content and protein kinase G (PKG) activity found in HFpEF patients contributes to a shortage in myocardial oxygen supply [25]. Accordingly, based on these processes, H-FABP might prove a promising tool in the diagnosis and controlling the success of treatment of HFpEF patients, quantitating the amount of subclinical ischemia. 

Regarding levels of sST2 we found a significant increase in ICM and DCM patients compared to controls and HFpEF, while no significant difference between HFpEF patients and the control group was observed. There are two isoforms of ST2, which both act as receptor to Interleukin-33: The membrane bound ST2L receptor responsible for potential cardioprotective effects, mediated trough IL-33 and the soluble ST2, which acts as a decoy receptor for IL-33 [26]. Due to its role as decoy receptor for the cardioprotective IL-33, sST2 constitutes a marker of increased cardiac strain and cardiac fibrosis and was also reported to be elevated in inflammatory diseases [26,27]. Moreover, studies have shown increased levels and a prognostic relevance of sST2 in HFrEF and acute coronary syndrome [14]. Accordingly, our findings regarding elevated concentrations of sST2 in ICM and DCM patients are consistent with former studies. However, contrary to our expectations, HFpEF patients evidenced low levels of sST2 similar to the control group. This finding also matches former studies, which reported lower levels of sST2 in HFpEF compared to HFrEF [28]. Further and bigger studies are required to verify these findings and help in explaining the underlying mechanisms of these results. Nevertheless, the process of fibrosis itself represents an important prognostic factor also for HFpEF patients [29]. Thus, despite the low levels, sST2 could potentially serve as monitoring parameter in HFpEF analogical to its application HFrEF patients due to the representation of fibrosis progression.

Similar to our findings on sST2, we found significantly elevated levels of suPAR in ICM and DCM patients compared to controls and HFpEF, while again no significant differences were observed between HFpEF patients and controls. The membrane bound uPAR is mainly expressed on the cell membrane of immunocompetent cells [30]. The soluble form (suPAR) is created through the cleavage and release of uPAR [30]. Correspondingly, suPAR represents a marker of inflammation and immune system activity [30,31]. A significant correlation of suPAR with myocardial infarction and HFrEF has been demonstrated [10,11]. The finding of increased suPAR levels in ICM and DCM patients might be mainly explained by a higher prevalence of inflammatory processes present in HFrEF, also triggered by further concomitant diseases. Further, especially larger studies should be performed to scrutinize for an explanation of these findings. To further analyze the diagnostic implications of biomarkers in HFpEF patients, we conducted an AUC analysis. Here we found considerably high values for GDF-15 and H-FABP in contrast to sST2 and suPAR. Additionally, to further evaluate the diagnostic potential of biomarkers in HFpEF patients, we conducted a pairwise comparison of ROCs. This further confirmed our previous findings of H-FABP and GDF-15 constituting paramount diagnostic markers for HFpEF. In contrast, sST2 and suPAR did not seem to have a major diagnostic benefit (see Table 2). Accordingly, with regards to HFpEF patients, GDF-15 and H-FABP represent the most promising markers for the future. 

All biomarkers included in our study evidenced a significant correlation with creatinine, BNP and CRP as well as an inverse correlation with ejection fraction. Most importantly, the highly significant correlation with BNP and ejection fraction emphasizes their great potential as heart failure biomarkers. However, contrary to BNP, which is mainly secreted by cardiomyocytes in response to volume increase, novel biomarkers are involved in numerous different pathophysiologic processes, thus providing additive information to natriuretic peptides. These processes comprise subclinical ischemia and ischemic events (H-FABP) as well as cardiovascular remodeling and inflammatory processes (sST2, GDF-15 and suPAR) [11,12]. Since all these processes represent key factors in the development and progression of heart failure, novel biomarkers offer a promising opportunity to assess the impact of comorbidities on this regard [3,4]. Correspondingly, the involvement of novel biomarkers in inflammatory processes was also observed in our study, reflected by a significant correlation of all markers with CRP. In addition to novel biomarkers tested in our project, latest studies also proposed an analysis of micro-RNA expression patterns as a novel diagnostic approach in heart failure [32,33,34]. On this regard, De Rosa et al. could show, that transcoronary concentration gradients of circulating microRNAs could help to distinguish between different heart failure entities [33]. Similar to biomarkers in our study, circulating and exosomal micro-RNAs were also shown to correlate with clinical parameters such as left ventricular function in former studies [32,34]. In consequence, micro-RNA analysis might offer a great diagnostic benefit in the assessment of heart failure in the future. Moreover, micro-RNAs were also shown to provide diagnostic potential in other cardiovascular diseases as for example coronary artery disease and myocardial infarction [32,34]. However, while standardized testing kits for a clinical application of novel biomarkers are already available and their application is also represented in current guidelines, the diagnostic application of micro-RNA testing has yet to be implemented in clinical practice.

With regards to our findings, suggestions on the future role of H-FABP and GDF-15 in HFpEF are highly speculative due to the hypothesis generating character of our study. Nevertheless, since established testing kits are already available, their use in addition to already established markers such as BNP might be a useful approach for the future. Especially with regards to the pathophysiology in HFpEF, a combination of natriuretic peptides and novel markers seems reasonable, in order to target the different processes involved in this disease [9,19,23]. Taken together, novel biomarkers represent a promising diagnostic approach in HFpEF patients. Based on their expression patterns, they reflect different pathophysiological processes relevant in this disease entity and thus might enable a more precise diagnosis of HFpEF in the future.

## 5. Conclusions

In summary, novel cardiovascular biomarkers provide a considerable potential to add to the diagnostic process in HFpEF patients. While sST2 and suPAR did not show a relevant dynamic in HFpEF patients compared to controls, a significant difference was evident for H-FABP and GDF-15. These findings point towards a relevant role of subclinical ischemia in HFpEF patients and offer a new aspect in this complex pathophysiology. The increase in GDF-15 might be mainly induced by myocardial remodeling and fibrosis. Thus, GDF-15 could also offer a prognostic benefit in the future. However, cardiac biomarkers showed a lower overall expression in HFpEF patients compared to other heart failure entities, emphasizing the diagnostic challenges in HFpEF. Nevertheless, by combining the information of different pathophysiological processes by means of a multi-marker approach, novel biomarkers might be very useful in the identification of HFpEF patients in the future. 

## 6. Limitations

The most important limitation of our study is the small sample size of the HFpEF cohort involved. This of course markedly limits the results of the current analyses. Moreover, the diagnostic criteria for HFpEF is a matter of ongoing debate and represents a clinical challenge as already mentioned above. Accordingly, the findings of the study must be interpreted with care. Additionally, the single-center and retrospective character must be taken into account. As no follow-up was performed, the dynamic of biomarkers in the progression of heart failure cannot be reflected. Moreover, our study does not include a comparison with already established markers as for example BNP. In consequence, direct comparison is limited. Despite the limitations mentioned above, the present study points out the potential benefits and advantages of the application of novel biomarkers in the diagnosis of heart failure and HFpEF. As our study suggests a diagnostic benefit in HFpEF patients, our results give rise to further investigation.

## Figures and Tables

**Table 1:** Baseline Characteristics.

	Controls		HFpEF		ICM		DCM		Total		*p*-Value
	Mean	SD	Mean	SD	Mean	SD	Mean	SD	Mean	SD	
Age (y)	63.56	9.25	70.94	6.49	65.12	11.16	57.10	10.73	62.65	10.73	<0.0001
Height (m)	1.68	0.09	1.69	0.09	1.74	0.09	1.75	0.09	1.72	0.09	<0.0001
Weight (kg)	77.68	17.22	81.86	12.82	76.66	25.58	89.09	18.74	81.06	20.27	0.001
BMI	27.22	5.71	28.68	4.63	28.08	4.37	29.02	5.24	28.30	5.08	0.334
LVEF (%)	65.93	8.63	59.75	9.85	37.42	12.93	35.32	11.87	48.04	17.92	<0.0001
BNP (pg/mL)	73.74	86.08	165.22	162.54	435.75	488.22	684.64	866.83	430,10	646.27	<0.0001
Creatinine (μmol/L)	74.29	15.67	85.06	25.96	108.19	39.05	98.35	31.15	89.14	30.44	<0.0001
GFR (mL/min)	83.62	13.33	71.08	13.78	66.97	17.61	74.69	24.96	75.75	17.68	0.084
CRP (mg/L)	2.28	2.98	5.58	8.87	4.33	4.20	7.55	14.29	4.55	9.19	0.005
Hb (mmol/L)	8.79	0.56	8.16	0.87	8.51	0.94	8.92	0.91	8.67	0.91	0.005
LDL (mmol/L)	3.47	0.94	2.76	1.40	2.23	0.89	2.88	1.07	3.10	1.10	<0.0001
HDL (mmol/L)	1.49	0.41	1.29	0.35	0.99	0.22	1.15	0.31	1.32	0.40	<0.0001

**Table 2:** Concomitant diseases and medication.

	Controls	HFpEF	ICM	DCM	Total	*p*-Value
Sex (male)	36%	44%	86%	77%	61%	<0.0001
Diabetes	15%	39%	36%	38%	29%	0.003
Hypertension	77%	89%	78%	50%	70%	<0.001
Atrial Fibrillation	5%	50%	3%	18%	15%	<0.0001
Beta Blockers	39%	72%	100%	99%	76%	<0.0001
ACE-Inhibitors	59%	72%	96%	96%	82%	<0.0001
Loop-Diuretics	30%	56%	79%	91%	64%	<0.0001
MRA	2%	19%	61%	68%	43%	<0.0001

**Table 3:** Levels of biomarkers.

	Controls		HFpEF		ICM		DCM	
	Median	Interquartile Range	Median	Interquartile Range	Median	Interquartile Range	Median	Interquartile Range
sST2 (pg/mL)	4999.00	2970.00	4318.00	2332.00	7869.00	5191.00	7010.00	5892.00
GDF-15 (pg/mL)	561.20	276.60	838.00	415.90	720.50	565.60	639.10	595.10
H-FABP (ng/mL)	0.00	0.60	0.82	0.53	1.66	3.59	1.94	1.83
suPAR (pg/mL)	2414.00	1280.00	2279.00	1753.00	3576.00	2567.00	3280.00	2349.00

**Table 4:** AUC-Analysis.

Variable	AUC	SE ^a^	95% CI ^b^
ST2	0.567	0.0725	0.470 to 0.660
suPAR	0.543	0.0829	0.447 to 0.637
GDF15	0.787	0.0469	0.700 to 0.859
HFABP	0.792	0.0401	0.705 to 0.862

^a^ DeLong et al., 1988; ^b^ Binomial exact

**Table 5:** Pairwise comparison of ROC curves.

**ST2 ~ suPAR**	
Difference between areas	0.0240
Standard Error ^a^	0.112
95% Confidence Interval	−0.196 to 0.244
Z statistic	0.214
Significance level	*p* = 0.8307
**ST2 ~ GDF15**	
Difference between areas	0.220
Standard Error ^a^	0.0999
95% Confidence Interval	0.0247 to 0.416
Z statistic	2.207
Significance level	*p* = 0.0273
**ST2 ~ HFABP**	
Difference between areas	0.225
Standard Error ^a^	0.0830
95% Confidence Interval	0.0621 to 0.388
Z statistic	2.708
Significance level	*p* = 0.0068
**suPAR ~ GDF15**	
Difference between areas	0.244
Standard Error ^a^	0.0996
95% Confidence Interval	0.0492 to 0.440
Z statistic	2.453
Significance level	*p* = 0.0141
**suPAR ~ HFABP**	
Difference between areas	0.249
Standard Error ^a^	0.0983
95% Confidence Interval	0.0562 to 0.442
Z statistic	2.531
Significance level	*p* = 0.0114
**GDF15 ~ HFABP**	
Difference between areas	0.00439
Standard Error ^a^	0.0563
95% Confidence Interval	−0.106 to 0.115
Z statistic	0.0779
Significance level	*p* = 0.9379

^a^ DeLong et al., 1988.

## Supplementary Materials

The following are available online at https://www.mdpi.com/2077-0383/9/4/1130/s1, Table S1: Correlation Analysis of baseline characteristics and biomarkers; Table S2: Results after correction for multiple testing (Bonferroni-Holm).

## Abbreviations

AUC	area under the curve
BMI	body mass index
BNP	brain natriuretic peptide
CKD-EPI	Chronic Kidney Disease Epidemiology Collaboration
CRP	C-reactive protein
DCM	dilative cardiomyopathy
ESC	European society of cardiology
ELISA	enzyme-linked immunosorbent assay
GDF-15	growth differentiation factor-15
GFR	glomerular filtration rate
Hb	haemoglobin
H-FABP	heart-type fatty acid binding protein
HFpEF	heart failure with preserved ejection fraction
HFrEF	heart failure with reduced ejection fraction
HDL	high density lipoprotein
ICM	ischemic cardiomyopathy
IL-33	interleukin 33
LDL	low density lipoprotein
LVEF	left ventricular ejection fraction
MRA	mineralocorticoid receptor antagonist
NYHA	New York heart association
PKG	protein kinase G
ROC	receiver operating curve
sST2	soluble suppression of tumorigenicity 2
suPAR	soluble urokinase-type plasminogen activator receptor
